# RETRACTION: Synthesis, Characterization, and Anticancer Activity of New Quinazoline Derivatives against MCF‐7 Cells

**DOI:** 10.1155/tswj/9828625

**Published:** 2026-06-02

**Authors:** The Scientific World Journal

RETRACTION: F. L. Faraj, M. Zahedifard, M. Paydar, C. Y. Looi, N. A. Majid, H. M. Ali, N. Ahmad, N. S. Gwaram, M. A. Abdulla, “Synthesis, Characterization, and Anticancer Activity of New Quinazoline Derivatives against MCF‐7 Cells,” *The Scientific World Journal*, 2014, 212096, 10.1155/2014/212096

The above article, published online on 04 December 2014 in Wiley Online Library (wileyonlinelibrary.com), has been retracted by agreement between the the Academic Editor, Thomas Efferth; and John Wiley & Sons Ltd. The retraction has been agreed due to concerns identified with Figure 11a on PubPeer [1]. Further investigation by the publisher identified additional concerns with Figure 12a. A summary of the concerns is as follows:

• Figure 11a is identical to Figure 3a in [2]
• The control panels in Figure 12a appear identical to the Control panels shown in the correction posted in [3]

No response was received from the authors regarding the concerns raised. After assessment by the Editorial Board, the results and conclusions presented can no longer be considered reliable, and the article is therefore retracted.

The authors did not respond to the retraction.
